# The accumulation of rare-earth yttrium ions by *Penicillium* sp. ZD28

**DOI:** 10.1186/s13568-020-0961-8

**Published:** 2020-02-03

**Authors:** Weiying Wang, Chenglong Xu, Yiqiao Jin, Zhibin Zhang, Riming Yan, Du Zhu

**Affiliations:** 1 Key Laboratory of Protection and Utilization of Subtropical Plant Resources of Jiangxi Province, College of Life Sciences, Jiangxi Normal University, Nanchang, 330022 China; 2 Key Laboratory of Bioprocess Engineering of Jiangxi Province, College of Life Sciences, Jiangxi Science and Technology Normal University, Nanchang, 330013 China

**Keywords:** Rare earth yttrium ion, *Penicillium* sp. ZD28, Accumulation effect

## Abstract

To obtained fungal resources with excellent tolerance and accumulation capacity to rare earth yttrium ions (Y^3+^), rare earth ore samples were collected and used for microbial screening. A fungus hyper-resistant to Y^3+^ was obtained and the effects of the fungus in three physiological states (growth process, mycelial pellets with physiological activity and the fungus powder after being ground) on the Y^3+^ accumulation were investigated. The Y^3+^ resistant fungus was identified as *Penicillium* sp. ZD28, and its mycelium pellets (about 1 mm in diameter) showed poor ability to accumulate Y^3+^ with an adsorption capacity of less than 81 μmol/g. However, the fungus was able to remove 99% of Y^3+^ during the growth process, at an initial concentration of less than 600 μM. Bioaccumulation of Y was observed on the cell surface of the ZD28 strain by elemental mapping using scanning electron microscopy-energy dispersive X-ray spectroscopy. The adsorbent (the dry fungal powder) had a remarkable adsorption property for Y^3+^ that was greater than 455 μmol/g in conditions of 465 μM < [Y^3+^] < 6382 μM. *Penicillium* sp. ZD28 has major potential applications in the accumulation of yttrium group rare earth ions. This research has formed a theoretical foundation for the application of this biological method to extract rare earth ions in the mining and smelting of yttrium group rare earth elements.

Rare earth elements (REEs) comprise 17 elements in the periodic table, including 15 lanthanide elements, scandium (Sc) and yttrium (Y). Due to its unique physical and chemical properties, REEs are widely used in various fields of modern industry, particularly in many clean energy technologies and consumer products (Stone 2009; Alonso et al. 2012). In 2 decades, the price of REEs has been increasing, leading to the high prosperity in rare earth mining (Chen et al. 2013).

China has one of the most abundant REE resources in the world, with ion-adsorption heavy rare earth minerals accounting for two-thirds of China’s rare earth resources which provides greater than 90% of the global REEs supply (Wang 2014). “Foot Cave”, located in Longnan, Ganzhou city of Jiangxi province, is currently the largest ion-adsorption type heavy rare earth deposits. It covers an area of 40 km^2^ and is the main heavy earth element raw materials supplier in China. Rare earth ions are adsorbed on the surface of clay minerals, such as montmorillonite, kaolinite and muscovite. The yttrium group is the main element in clay $\left( {\sum {\text{Y}}_{ 2} {\text{O}}_{ 3} /\sum {\text{TR}}_{ 2} {\text{O}}_{ 3} \, \ge \, 90\% } \right)$ (Yang 2015). Five REEs (Tb, Dy, Eu, Nd, and Y) in particular have been highlighted by the U.S, because its critical to the development of ‘clean’ emerging energy technologies (Zhuang et al. 2015; US Department of Energy 2011). Huge market demand has led to a boom in yttrium-rich rare-earth mining. At present, in situ or pool leaching using ammonium sulphate solution is the main mining technology (Moldoveanu and Papangelakis 2012), however, this approach leads to serious environment problems, reflected in the production of large amounts of ammonia nitrogen waste-water (concentrations as high as 3500–4000 mg/L) during the course of leaching and extraction. A large quantity of ammonia nitrogen infiltrates into soil, groundwater and surface water along with rainfall. This results in a major excess of ammonia nitrogen in the water system causing a great threat to ecological security due to water eutrophication (Feng et al. 2017; Gao and Zhou 2011). Therefore, there is an urgent need to develop green extraction technology for applications in ion-adsorption rare earth mines (Xiao et al. 2015).

Microbial leaching technology is new metallurgical technology of mineral resources that is attracting increasing attention (Brierley and Brierley 2013; Kücüker et al. 2016). For ion-adsorption rare element mine, microbial mining is simpler as it involves biosorption (bio-accumulation). Microbially mediated surface adsorption (biosorption) represents a potentially cost-effective and eco-friendly approach for metal recovery (Li and Tao 2015; Zhuang et al. 2015; Moriwaki and Yamamoto 2013). Bacteria exhibit high metal adsorption capacities because of their large specific surface area, small volume and abundance of cell surface functional groups (e.g., carboxylates and phosphates) with metal coordination functionality (Moriwaki and Yamamoto 2013). Moreover, fast reproduction rates can increase the advantages for applications in rare earth extraction processes (Tsuruta 2007; Mullen et al. 1989). Compared to bacteria, large-sized fungal mycelia are more advantageous in environmentally friendly green technology. Furthermore, fungi can secrete more extracellular polymers which can significantly increase the yield of biosorption (Das 2010).

Currently, investigations into the microbial accumulation of rare-earth ions have mainly focused on bacterial biomass adsorption. In these studies, prepared bacteria were used for rare earth adsorption. For example, the adsorption capacities of bacterial powder made from *Pseudomonas aeruginosa* to La^3+^, Eu^3+^ and Yb^3+^ were 397, 290 and 326 µmol/g, respectively (Texier et al. 1999). However, metal ion accumulation during the growth of fungi has greater potential according to the studies by Horiike who found that the fungus *Penidiella* sp. T9, could efficiently accumulate dysprosium ions (Dy^3+^). They also showed that the Dy content in the cell pellet of the T9 strain was 910 µg/mg of dry cells (Horiike and Yamashita 2015), indicating that the accumulation capacity of Dy^3+^ was as high as 5400 µmol/g, which is significantly superior to that of *P. aeruginosa*. However, there are very few reports on adsorption of yttrium group rare-earth ions by fungi, which greatly hinders the application of biological methods in extraction and recovery of yttrium from “Foot Cave” mines and other ion-adsorption type heavy rare-earth deposits in the south of China.

In this study, soil samples were collected from the “Foot Cave” mine, which is a typical yttrium-rich rare earth ore, and a fungus with excellent tolerance and accumulation ability of Y^3+^ could be obtained by strain screening. The ability of the fungus in three physiological states (growth process, the mycelial pellets with physiological activity and the dry fungal powder after being ground) to accumulate yttrium was investigated. This study established a basic theory for the application of rare earth ion accumulation using fungi in ion-adsorption type REEs exploiting and provides a new concept for alleviating ecological destruction and environmental pollution from mining.

## Materials and methods

### Collection of soil from the “Foot Cave” and analysis of rare earth ions in soil samples

Early spring is the most suitable season for microbial reproduction. Topsoil was collected from the “Foot Cave” rare earth mine, from nine sampling points across mined (E114° 50′ 13.12″, N24° 50′ 20.47″) and unmined sites (E114° 50′ 12.67″, N24° 50′ 02.21″) in April. Three samples were taken from each site, and a total of 27 soil samples were taken back to the lab within 12 h. The samples were divided into two parts: one sample was placed at 4 °C for microbial screening, the other sample was air-dried and passed through a 200 mesh sieve after grinding and mixing, and then sent to the ALS Minerals laboratory (Guangzhou, China) to determine the content of REEs by ICP-MS/MS.

### Isolation of Y^3+^-tolerant fungi

Soil samples obtained from the REE mines were mixed together (0.5 g per sample) for the screening experiment. Then, 13.5 g of the mixed sample was transferred into enrichment medium (Potato Dextrose Broth, PDB). The mixtures were incubated at 28 °C and stirring at 120 rpm overnight. 10 mL of the culture was then injected into a fresh enrichment medium, followed by incubation at 28 °C and stirring at 120 rpm overnight. Then, the culture was subjected to separation using a PDA agar plate to obtain single clones. A total of eleven different strains of filamentous fungi with different appearances were obtained. Spores were collected and spore suspensions were prepared for the Y^3+^-tolerance assay.

PDA plates with different concentrations of Y^3+^ were prepared as follows: 200 g of small pieces of fresh potato were boiled in 800 mL of deionized water until they became fluffy mashed potato. 20 g of glucose was added to the water whilst hot. The volume was fixed to 1 L after filtration using a gauze. The prepared medium was divided into five equal parts, and placed in five 250 mL flasks. 1.5% agar was added before sterilization at 121 °C for 15 min. The PDA medium was cooled to about 55 °C, different volumes of Y^3+^ stock solutions (sterilized by 0.22 µm filtration membrane) were added, shaken and poured into petri dishes for use in fungal culture after solidification. The final concentration of Y^3+^ was 10, 200, 400 and 800 mg/L, respectively. The eleven filamentous fungal spores (2 µL) were inoculated on the PDA plates with different concentrations of Y^3+^. Growth and morphological changes were observed after being cultured at 28 °C for 60 h.

### Sequencing of the 5.8S rRNA gene

Strain number 1 was cultured in PDB for 3 days. Cultured cells were harvested by centrifugation (12,000 g, 5 min, 4 °C), and washed twice in sterile saline. Genomic DNA was extracted from the disrupted cells according to Murmur’s method (Marmur 1961). The 5.8S rRNA gene fragments were amplified from the extracted genomic DNA by PCR using PrimeSTAR^®^ Max DNA Polymerase (TaKaRa Bio Inc., Shiga, Japan) and primers ITS1 (5′-TCCGTAGGTGAACCTGCGG-3′) and ITS4 (5′-TCCTCCGCTTATTGATATGC-3′) (Gang et al. 2015). The PCR conditions were as follows: an initial denaturation step at 98 °C for 5 min, followed by 30 cycles of 98 °C for 10 s, annealing at 57 °C for 5 s, and elongation at 72 °C for 1 min. The reactions were carried out in a Mastercycler thermal cycler (Eppendorf Co., Ltd., Tokyo, Japan). The PCR products were used as templates for direct nucleotide sequencing. The sequencing was performed by Sangon Biotech (Shanghai) Co., Ltd. The generated sequences were compared to other fungal RNA sequences in the GenBank database using BLAST (http://blast.ncbi.nlm.nih.gov/Blast.cgi) (Altschul et al. 1990). The isolated sequences and the GenBank sequences were aligned using Clustal W. A phylogenetic tree of the aligned sequence data was generated using Molecular Evolutionary Genetics Analysis (MEGA5.0) (Tamura et al. 2011), and the neighbor-joining method (NJ) (Saitou and Nei 1987). The percentages of replicate trees in which the associated taxa clustered together in the bootstrap test (1000 replicates) are shown next to the branches (Felsenstein 1985). The evolutionary distances were calculated using the maximum composite likelihood method (Tamura et al. 2004), and shown as the number of base substitutions per site.

#### Nucleotide sequence accession number

The sequence determined in this study was deposited in the DNA Data Bank of USA (https://www.ncbi.nlm.nih.gov/nuccore) under accession number MN503307.

### Interaction between yttrium and *Penicillium* sp. ZD28 during the growth process

The interaction between yttrium and *Penicillium* sp. ZD28 during the growth process was determined under culture condition using Czapek’s medium. Yttrium hydroxide precipitation can occur under conditions of pH > 5 (Tsuruta 2007), and some precipitation containing yttrium were produced with the increasing of Y^3+^ concentration. According to our preliminary experimental results, the initial pH of the medium was adjusted to 3.0, and so no precipitation would occur when the concentration of Y^3+^ was less than 600 µM. Czapek’s medium (pH 3.0) was sterilized at 115 °C for 30 min. Different volumes of Y^3+^ stock solutions (0.5 M, sterilized by 0.22 µm filtration membrane) were added to the medium after cooling. The spore suspension (10^8^/mL) was inoculated and cultured at 28 °C with stirring at 120 rpm for 3 days. In the control samples, the spore suspension was replaced by 1 mL of sterile water. The experiment was repeated four times in each group. At the end of culture period, the supernatant and mycelium were separated by suction filtration. The Y^3+^ concentration in the supernatant from the groups with and without fungal inoculation was measured, and the difference between them is the accumulation of Y^3+^ concentration by the growing fungus. The mycelium was dried to a constant weight at 50 °C.

After culturing at the different concentrations of Y^3+^, the cultures in the flask were poured into petri dishes to observe the surface morphology changes of *Penicillium* sp. ZD28. The feather-like mycelium in group with the highest Y^3+^ concentration were fixed for 1 h at room temperature in 2.5% glutaraldehyde containing Y^3+^ at a concentration approximately equal to the initial concentration in medium, and the cells were then washed free of glutaraldehyde with Y^3+^ solution (Mullen et al. 1989). The cell pellets were taken into coverslip, spray-gold after air-dry, then observed by scanning electron microscopy (SEM) (S-3400 N; Hitachi High-Technologies Corp., Tokyo, Japan) operated at 30 kV. Energy-dispersive X-ray spectroscopy (EDX) (Quantax70; Bruker AXS Microanalysis GmbH, Karlsruhe, DE) was used to acquire the X-ray spectra and map the elements.

At the same time, Czapek’s medium solid plates with the same concentration gradient of Y^3+^ as the liquid medium were prepared. 2 µL of spore suspension was added to the plates and a cover slide inserted obliquely near the fungus. The mycelium and spore morphology on the cover glass were observed by SEM after 3 days of culture.

### Accumulation of Y^3+^ by the mycelium pellet and the dry fungal powder

Preparation of the mycelium pellet: 100 mL of PDB medium was placed in 250 mL flask, and sterilized at 121 °C for 15 min. 1 mL of spore suspension with a concentration of 10^8^/mL was inoculated and cultured at 28 °C and stirred at 150 rpm for 3 days. The mycelium pellet was obtained by suction filtration with filter paper, and used for yttrium ion adsorption, immediately after washing three times in deionized water.

Preparation of the dry fungal powder: the mycelium was obtained using the same method described above, and then dried at 50 °C to a constant weight. The fungal powder was used for yttrium ion adsorption after grinding through a 100 mesh.

Accumulation of yttrium ions by the above two adsorbents: The appropriate amount of mycelium pellets could be dried at 50 °C to a constant weight, and the water content was calculated to be 13.35 (± 0.50)%. 11.54 (± 0.05) mg of the pellets (equal to 10 mg of dry fugal powder) and 10 (± 0.05) mg of the fungal powder were put into a 15 mL centrifuge tube. 10 mL of yttrium ion solution at different concentrations (about from 0.5 to 8.0 mM) was added and the pH adjusted to 5.0 with 6 M hydrochloric acid. The above reaction was repeated three times in each group, and the supernatant was obtained by centrifuging at 6000 rpm for 5 min after the equilibrium reaction for 6 h in a tube oscillator (Qilinbeier KB5010, Haimen, China) at room temperature. The Y^3+^ concentration in the supernatant was determined, which is denoted by C_e_. The concentrations form the different prepared yttrium ion solutions were determined, which is denoted by C_0_.

### Determination of Y^3+^ concentrations

ICP-MS/MS method: High concentrations of yttrium ions can cause precipitation formation in Czapek’s medium and so the accumulation of Y^3+^ by the growing fungus must be carried out in relatively low concentrations of yttrium ion. However, the concentration of yttrium ions was lower than the detection limit of the chemical method. Yttrium ion accumulation during growth of the fungus was detected using an ICP-MS/MS method (Agilent, 8900, USA). Yttrium concentrations of 0, 5, 10, 15, 20 ppb were used as a standard curve.

Arsenazo III colorimetric method (Hogendoorn et al. 2018): Y^3+^ adsorption by the mycelium pellets and the dry fungal powder was determined by the Arsenazo III colorimetric method. The reaction system was as follows: 1 mL of citric acid/phosphate buffer (pH 2.8), 980 µL of the sample and 20 µL of 1 mM Arsenazo III. A UV754N spectrophotometer (Youke, Shanghai, China) was used to measure optical density at 650 nm. Yttrium concentrations of 0.5, 1, 5 and 10 mM were used to generate a standard curve.

## Results

### Content of rare earth element in ore samples

The content of rare earth ions in the soil samples showed very difference in content of light rare earth elements (LREEs) and heavy rare earth element (HREEs). Our results showed that HREEs are more abundant than LREEs both in exploited (sample 1–5) and unexploited (sample 6–9) soils. There was no significant difference in LREEs content between in the exploited and unexploited soil. However, the content of HREEs in the unexploited samples was around an order of magnitude higher than that in the exploited soils. It is worth highlighting that the Y^3+^ content was highest in unexploited soil (surface soil) which reached up to an average of 1323 mg/kg (Table 1).

**Table 1:** Light and heavy rare earth element content in soil samples

Soil samples^a^	Content of the light REEs (mg/kg)					
	La	Ce	Pr	Nd	Sm	Eu
1	20.4 ± 1.5	42.7 ± 10	8.70 ± 2.1	44.7 ± 3.5	33.6 ± 11.2	0.13 ± 1.5
2	1.3 ± 0.3	29.5 ± 5.0	0.94 ± 0.2	5.2 ± 1.5	6.06 ± 2.0	0.03 ± 0.01
3	14.7 ± 3.9	28.5 ± 1.2	4.58 ± 1.5	20.2 ± 5.2	5.10 ± 1.4	1.85 ± 1.5
4	111.0 ± 8	98.6 ± 6.0	30.1 ± 2.6	118.5 ± 51	24.4 ± 1.8	6.03 ± 1.2
5	19.8 ± 2.3	50.1 ± 3.2	8.89 ± 1.5	44.2 ± 5.6	32.8 ± 11.0	0.20 ± 0.1
6	20.8 ± 1.5	40.1 ± 6.2	8.52 ± 2.1	47.7 ± 2.9	40.4 ± 10.2	0.58 ± 0.12
7	20.8 ± 5	43.5 ± 14.1	8.46 ± 1.5	46.0 ± 11.2	39.8 ± 5.3	0.49 ± 0.23
8	16.1 ± 3.2	31.1 ± 2.3	6.77 ± 1.1	37.8 ± 9.0	33.3 ± 5.0	0.45 ± 0.33
9	20.5 ± 2.0	34.7 ± 10.1	8.19 ± 3.9	45.5 ± 5.1	41.1 ± 11.2	0.52 ± 0.12

Soil samples^a^	Content of the heavy REEs (mg/kg)								
	Gd	Tb	Dy	Ho	Er	Tm	Yb	Lu	Y
1	61.6 ± 11.0	12.45 ± 2.1	88.3 ± 11.2	18.95 ± 2.1	58.7 ± 2.0	8.43 ± 5.1	60.0 ± 21.5	9.40 ± 2.1	624 ± 101.0
2	13.60 ± 8.5	3.66 ± 1.5	30.7 ± 10.2	7.87 ± 1.5	27.3 ± 1.6	4.90 ± 2.0	39.1 ± 3.6	6.46 ± 1.5	173.5 ± 89.2
3	6.31 ± 1.5	0.95 ± 0.2	6.14 ± 1.2	1.25 ± 0.6	3.47 ± 1.8	0.45 ± 0.23	2.92 ± 2.1	0.43 ± 0.2	36.3 ± 10.0
4	22.1 ± 3.5	2.96 ± 0.3	16.70 ± 6.2	3.31 ± 1.2	8.36 ± 2.3	1.06 ± 0.56	6.68 ± 0.56	1.02 ± 0.2	84.5 ± 12.3
5	59.7 ± 12.3	13.60 ± 2.3	100.5 ± 20.1	22.7 ± 2.3	73.9 ± 21.0	11.70 ± 2.3	85.6 ± 18.2	13.40 ± 1.8	633 ± 80.7
6	108.5 ± 20.0	21.6 ± 4.2	153.5 ± 20.0	35.3 ± 12.4	109.5 ± 36.8	15.75 ± 6.5	101.5 ± 25.6	16.65 ± 8.3	1415 ± 500.1
7	105.0 ± 18.0	21.1 ± 5.2	153.5 ± 18.3	35.0 ± 14.2	107.5 ± 50.2	14.90 ± 4.5	100.0 ± 35.1	16.25 ± 4.0	1405 ± 436.1
8	87.9 ± 2.3	17.40 ± 3.9	125.5 ± 17.2	28.8 ± 10.1	88.7 ± 12.5	12.60 ± 7.2	85.7 ± 25.3	14.00 ± 2.9	1095 ± 221.0
9	108.5 ± 14.2	21.8 ± 4.8	156.0 ± 26.3	35.1 ± 9.8	107.0 ± 41.2	14.75 ± 6.3	97.6 ± 38.1	15.65 ± 5.6	1380 ± 400.1

^a^Samples 1–5 were from the mined spot, where the soil is high in quartz and gray-white in color. Samples 6–9 were obtained from the unmined spot, where the soil is red topsoil after removing withered leaves

### Isolation of Y^3+^-tolerant microorganisms

**Fig. 1:**
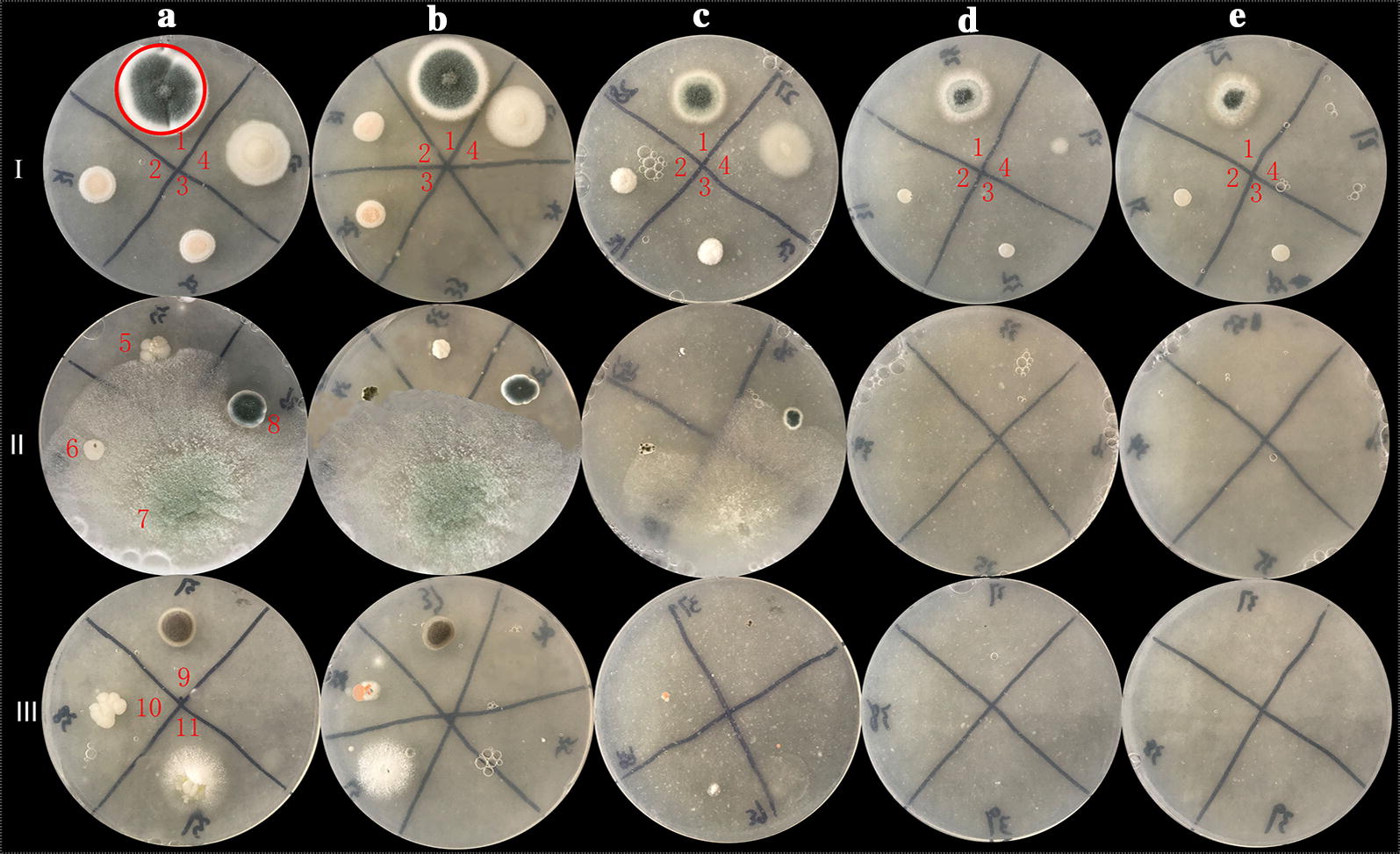
Effects of different concentrations of Y^3+^ on fungal growth. The concentration of Y^3+^ in column of **a**–**e** were 10, 100, 200, 400 and 800 mg/L, respectively; the line I, II and III contain strains number 1–4, 5–8, 9–11, respectively. The strain number 1 in red circle is the target fungus, identified *Penicillium* sp. ZD28

**Fig. 2:**
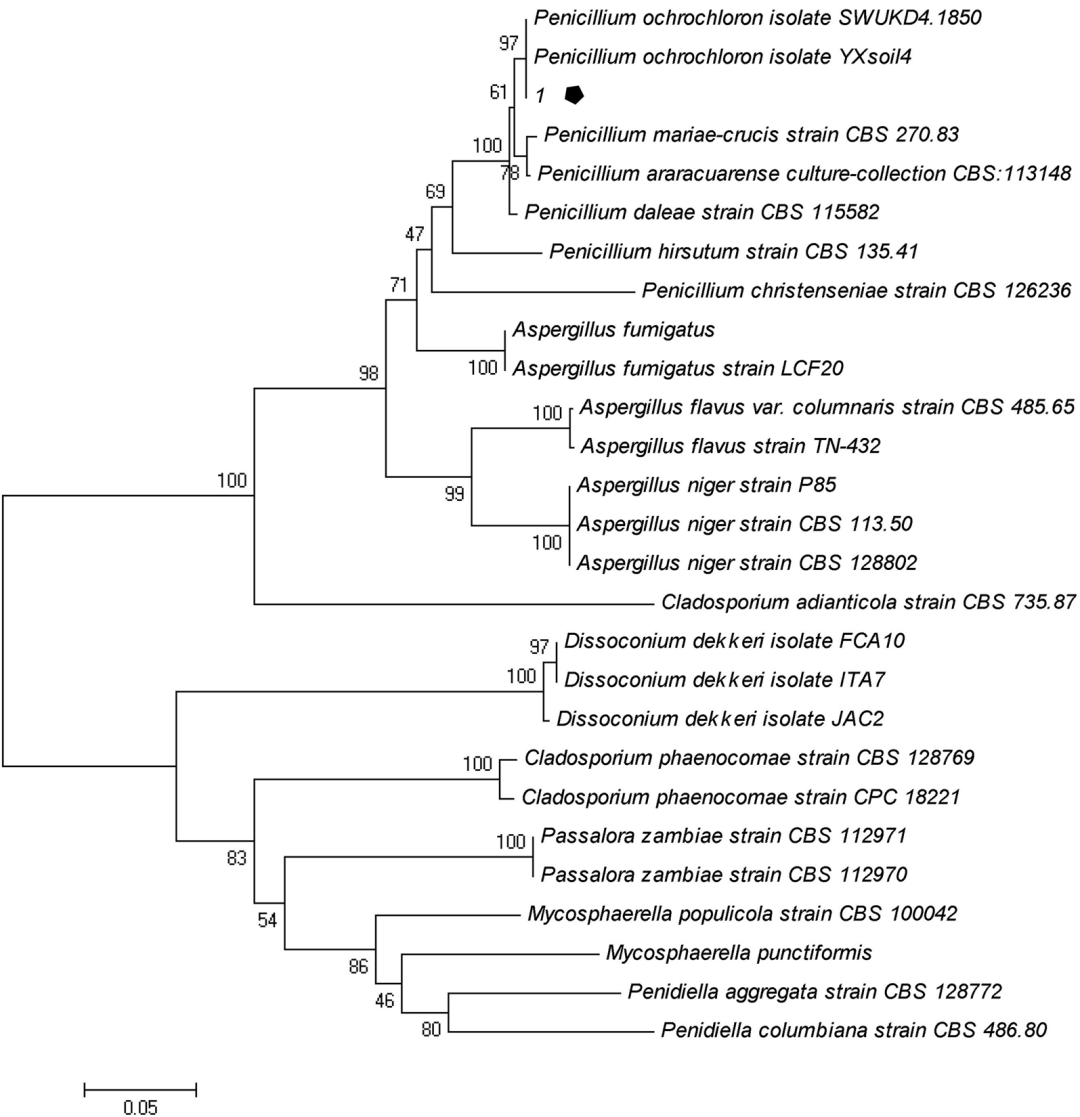
Phylogenetic tree inferred from the 5.8S-ITS1/ITS4 rRNA gene sequences of *Penicillium* and *Aspergillus*

Eleven fungi strains were obtained by preliminary screening. The eleven isolates were inoculated on PDA plates with different concentrations of Y^3+^. The morphology and size of colonies were different under different concentrations of Y^3+^ over a cultivation period of 3 days (Fig. 1). The colonies gradually became smaller with increasing Y^3+^ concentration. Strains numbers 1 to 4 were able to live at 800 mg/L Y^3+^, indicating a high tolerance to Y^3+^. Amongst these strains, strain number 1 had the largest tolerance to Y^3+^. The colony size gradually decreased with the increase of Y^3+^ concentration. However, the mycelium micromorphology and spore production were not affected, showing that the fungus had a high tolerance to Y^3+^. Therefore, strain number 1 was used as the target strain in this study. Also, the highest tolerated concentration of Y^3+^ in fungus samples 7 and 8 was 200 mg/L, and that of strains number 5 and 6 was 100 mg/L under solid culture conditions. A partial nucleotide sequence (554 bases) of the ITS1/ITS4 rRNA gene of the strain number 1 was determined and compared with sequences in GenBank using BLAST. The nucleotide sequence was found to have 99.8% homology with *P. ochrochloron* YXsoil4 (MH128152.1), and 99.1% homology with *P. ochrochloron* SWUKD4.1850 (KX346178.1). These fungi are classified as class *Plectomycetes*, order *Onygenales*, family *Eurotiaceae*, genus *Penicillium*. The phylogenetic tree (Fig. 2) shows high similarity between strain number 1 and the *P. ochrochloron*. Therefore, strain number 1 was named *Penicillium* sp. ZD28 together with the microscopic characteristics. The fungus has been deposited in the China Typical Model Cultivation Center with preservation number CCTCC M 2019865.

### Interactions between *Penicillium* sp. ZD28 and Y^3+^

**Fig. 3:**
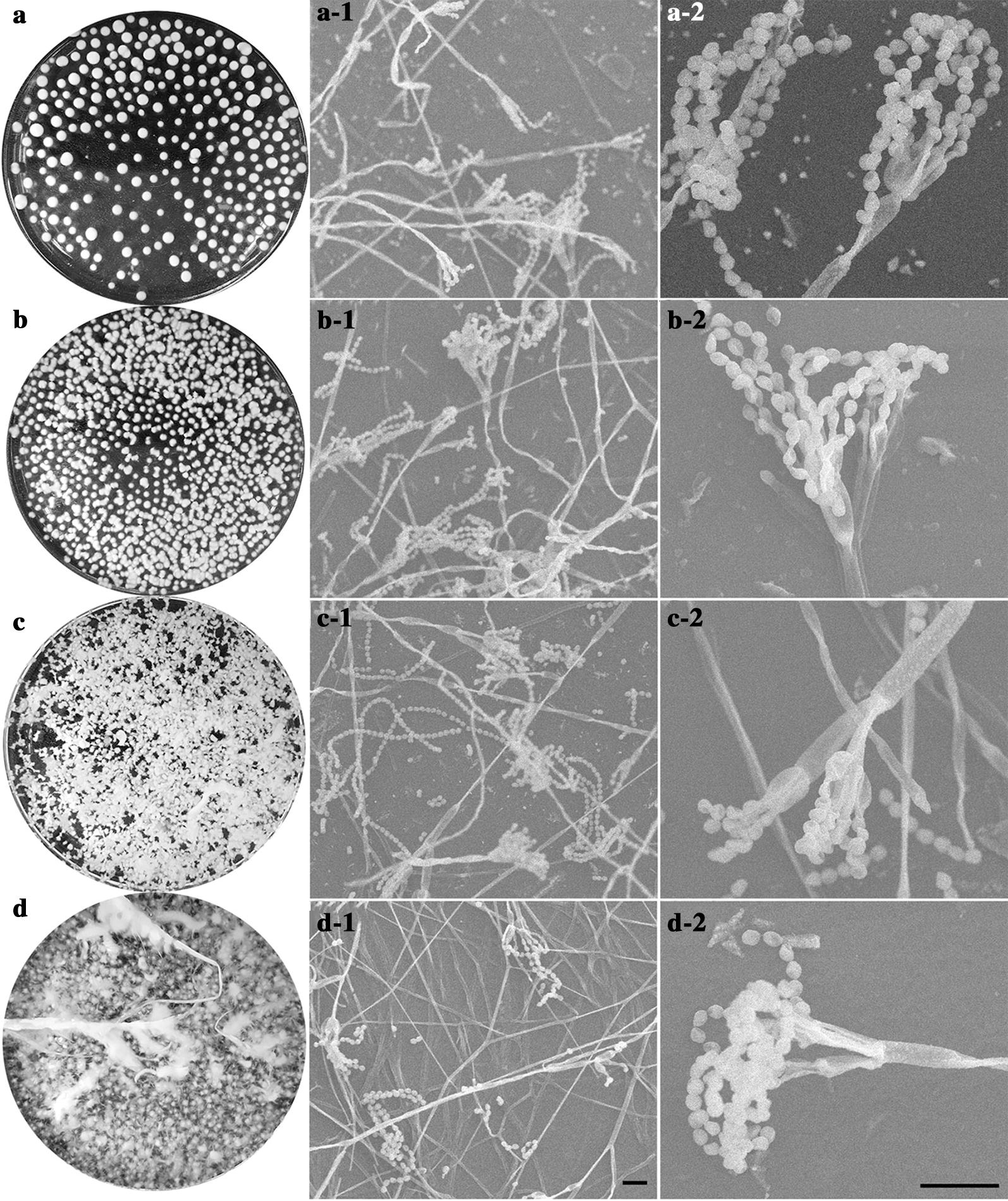
Effects of different concentrations of Y^3+^ on the morphology of *Penicillium* sp. ZD28. **a**–**d** are the mycelium pellet morphologies of *Penicillium* sp. ZD28 under the culture conditions with different concentrations of Y^3+^ for 3 days. **a**-**1**/**2**, **b**-**1**/**2**, **c**-**1**/**2** and **d**-**1**/**2** denote micromorphologies of *Penicillium* sp. ZD28 cultured with different concentrations of Y^3+^, scale bar  10 μm. The concentrations of Y^3+^ added according to the **a**/**-1**/**-2**, **b**/**-1**/**-2**, **c**/**-1**/**-2**, **d**/**-1**/**-2**, were 0, 73.71, 461.57, 580.67 μM respectively.

**Fig. 4:**
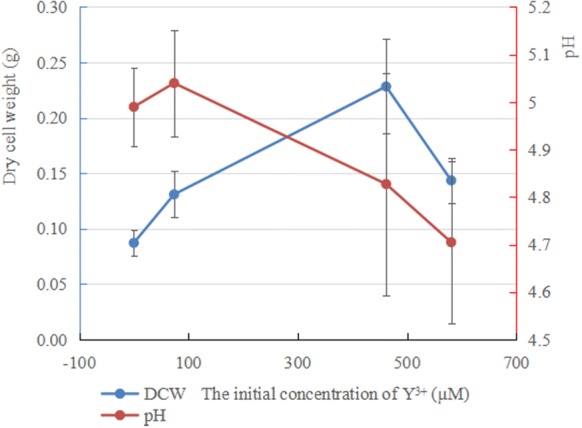
The effect of different concentrations of Y^3+^ on the mycelium biomass and pH of cultures

**Fig. 5:**
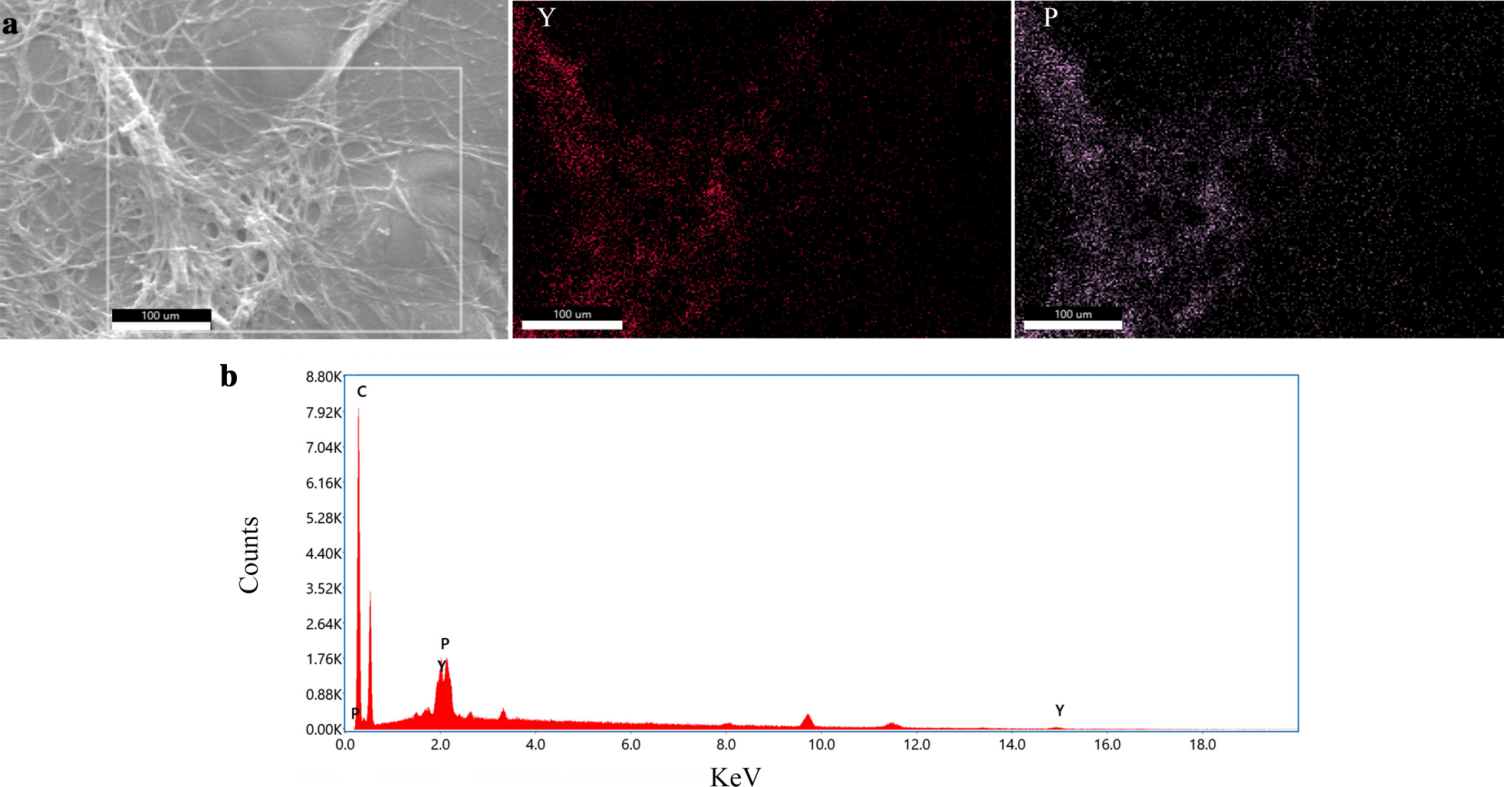
SEM-EDX analyses of Y in the ZD28 strain. **a** Back scattered electron image of the ZD28 strain after cultivation in the presence of Y (left) and elemental maps of Y(red, middle) and P (pink, right). **b** Scans of the white rectangle box in **a**

Yttrium has a major influence on the morphology of *Penicillium* sp. ZD28 under liquid culture conditions: yttrium at low concentration promotes an increase in fuangal biomass whilst yttrium at high concentration inhibits biomass. When Y^3+^ was added into the Czapek’s medium at concentration of 73.71 μM Y^3+^, the dry weight of mycelium pellets was significantly higher than that of in the absence of Y^3+^, but the shape of the mycelium pellets remained unchanged. When the concentration of Y^3+^ reached 461.57 μM, mycelium pellets were small and irregular in shape. The surfaces of the mycelium pellets were mostly radial and the dry weight of mycelium reached a maximum at this concentration (Fig. 4). When the concentration of Y^3+^ continued to rise to 580.67 μM, the shape of pellets became extremely irregular and seems to be feather-like. At this time, the dry weight of the mycelium decreased, but it was still higher in the absence of Y^3+^. These data indicated that Y^3+^ could promote the growth of mycelia across a certain concentration range, and yttrium at high concentration is toxic to the fungus. However, Y^3+^ had no effect on the micro-morphology and spore formation of *Penicillium* sp. ZD28 as observed with SEM (Fig. 3). In addition, there was no significant difference in pH between the groups at different concentrations of Y^3+^ (Fig. 4). In the SEM–EDX analyses, solidified Y was observed at the same location as P all over the cell surface (Fig. 5).

### Yttrium bioaccumulation by the *Penicillium* sp. ZD28

Analysis of the accumulation of Y^3+^ by the fungus during microbial growth found that the accumulation of Y^3+^ per gram of dry cells was different under different concentrations of Y^3+^ (Table 2). *Penicillium* sp. ZD28 accumulated 54.89 μmol/g of Y^3+^ when the initial yttrium concentration was 73.71 μM, and the removal rate was 97%. The fungus accumulated 199.07 μmol/g when the initial yttrium concentration was 461.57 μM, and the removal rate was 99%. The content of Y^3+^ in the fungus reached a maximum of 411.32 μmol/g when the initial yttrium concentration was 580.67 μM, and the removal rate was 99%.

**Table 2:** Accumulation of Y^3+^ during the growth process of *Penicillium* sp. ZD28

The initial concentration of Y^3+^ (µM)	Dry weight of the fungal biomass (g)	The residual concentration of Y^3+^ (μM)	The accumulation concentration of Y^3+^ (μmol/g)	Removal rate (%)
73.71 (± 10.02)	0.13 (± 0.02)	2.35 (± 1.27)	54.89	97
461.57 (± 23.29)	0.23 (± 0.04)	3.70 (± 0.57)	199.07	99
580.67 (± 34.51)	0.14 (± 0.02)	4.82 (± 0.59)	411.32	99

The accumulation effect of fungus adsorbent on Y^3+^ was determined by spectrophotometry and the results are summarized in Table 3. The accumulation content and removal rate of Y^3+^ by the active mycelium pellets were both significantly lower than that those by the microbial powders. With increasing initial concentration of Y^3+^, the adsorption capacity and remove rate of Y^3+^ by the active mycelium pellets gradually decreased, and the adsorption capacity of Y^3+^ by the dry fungal powders increased gradually, whilst the removal rate decreased gradually.

**Table 3:** Adsorption of Y^3+^ by the mycilium pellets and the dry fungal powder

C_0_ (µM)	The mycelium pellets			The fungal powder		
	Ce (µM)	S (µmol/g)	Removal rate (%)	Ce (µM)	S (µmol/g)	Removal rate (%)
465.19 (± 70.57)	386.25 (± 67.44)	78.94	17	10.01 (± 10.43)	455.18	98
1052.21 (± 78.07)	978.66 (± 57.04)	73.55	7	352.01 (± 30.40)	700.20	67
4804.36 (± 91.90)	4675.50 (± 88.30)	58.87	1	3983.81 (± 54.90)	821.55	17
6382.63 (± 134.88)	6360.56 (± 156.61)	22.08	0.3	5455.48 (± 104.32)	927.15	15

The fungal powder adsorption of Y^3+^ from solution was described well by the linearized Freundlich adsorption isotherm equation:

$$\mathop {\log }\nolimits_{10}^{S} \, = \,\mathop {\log }\nolimits_{10}^{K} \, + \,n\,\mathop {\log }\nolimits_{10}^{C}$$

where *S* is the amount of metal adsorbed in micromoles per gram, *C* represents the equilibrium solution concentration in micromoles per liter, and *K* and *n* are the Freundlich constants. The constant *K* represents the predicted quantity of metal removed in micromoles of metal per gram of dry cells at an equilibrium concentration of 1 μM. The simulation equation is as follows, y = 0.1056x + 2.5587, r^2^ = 0.9792. The Y^3+^ content removed by *Penicillium* sp. ZD28 is 362 μmol/g at an equilibrium concentration of 1 μM.

## Discussion

Ion-adsorption REEs are one of the most important rare earth mineral resources in China. There is a lack of new green technology of resource exploitation, which restricts development and utilization of ion-adsorption rare earth resources to some extent. Several studies have reported on microbial adsorption (accumulation) of rare-earth ions, however, most of studies focused on light and medium rare earths such as lanthanum, europium, samarium and dysprosium (Ozaki et al. 2015; Tsuruta 2006; Texier et al. 1999) whilst few have reported on heavy rare earths. Middle and heavy rare earth deposits in southern China account for two-thirds of the total reserves of rare earth deposits in China. In particular, the “Foot Cave” deposits in the south of Jiangxi province is yttrium-rich and yttrium accounts for more than 90% of the total rare earth content. Analysis of REE content in soil samples supported that “Foot Cave” as an yttrium-rich ion-adsorption type rare earth deposit. REEs in the granite parent rock are very low-grade. With the weathering of the minerals, the REEs migrate upwards and absorb onto the surface of clay minerals in the form of ions. Therefore, this study on the interactions between yttrium ion and fungus, which originated from the “Foot Cave”, is of great theoretical value and practical significance in the application of fungal adsorption of rare earth ions.

There have been many reports on the adsorption of rare earth ions by microbial powders. For example, the adsorption of rare earth lanthanum ions (La^3+^) by various bacteria (*Bacillus cereus*, *B. subtilis*, *Escherichia coli and P. aeruginosa*) was tested. The results showed that bacterial adsorption of La^3+^ conforms to the Freundlich model, and an average of 27% of the total La^3+^ was adsorbed from 1 mM solutions. Precipitates composed of lanthanum are crystalline, needlelike deposits that formed around the *P. aeruginosa* cells (Mullen et al. 1989). Seventy-six strains including bacteria, actinomycetes, fungi and yeast were used to adsorb the rare-earth element Sm. The results indicated that gram-positive bacteria exhibited a particularly high capacity for accumulation of Sm. In particular, *B. licheniformis* cells accumulated approximately 316 µmol Sm per gram dry wt. of microbial cells (Tsuruta 2007). However, there are very few reports on adsorption of yttrium group rare-earth ions by fungi, especially during the growth of microorganism. Tolerance has to be considered when we do the researches about microbial accumulation for yttrium ions during the growth process of fungi. When exposed to high concentration of Y^3+^, microorganism with good tolerance can be survival, growth and then adsorption even absorption. Therefore, the strain number 1 with the largest tolerance to Y^3+^ was designated as the target fungus. Luckily, the adsorption capacity for Y^3+^ was significant during the growth of *Penicillium* sp. ZD28, where the removal rate was around 99% when the initial concentration of Y^3+^ was less than 0.6 mM. Of course, there may be no direct correlation between tolerance and adsorption capacity, as reported by d’Aquino. He found that REE accumulation in *Trichoderma harzianum* T22 is lower than that in *T. atroviride* P1, although T22 has better tolerance. He also found that growth stimulation by REE and REE accumulation in fungal biomass are not directly related to each other (d’ Aquino et al. 2009). It is complicated that the relation between growth stimulation, fungal tolerance to REE and REE accumulation in fungal biomass.

In this study, the fungal powder from *Penicillium* sp. ZD28 showed good adsorption performance for Y^3+^. The adsorption capacity for Y^3+^ was greater than 455 µmol/g under initial concentrations of 0.4, 1.0, 4.8 and 6.4 mM, and higher than the previously reported microbial adsorption capacity of rare earth ions. These findings indicate that the fungal powder has great potential in ion adsorption of yttrium group rare earth. Also, low concentration yttrium can increase fungal biomass, whilst high concentrations can inhibit the growth and the number of spores (Fig. 1). Under liquid culture conditions, the mycelium exhibited a diffuse feathery appearance with increasing the concentration of Y^3+^, not due to the decrease in pH caused by the addition of Y^3+^ (Fig. 4), really because yttrium ions adsorbed on the surface of mycelium (Fig. 5) or entered the cell to inhibit the extension of mycelium. However, no obvious effect on the micromorphology of mycelium and spores was observed (Fig. 3).

Investigations into the accumulation of Y^3+^ by fungus in three physiological states (growth process, the mycelial pellets with physiological activity and the fungus powder after being ground) were conducted. Results showed that the prepared mycelium pellets (about 1 mm in diameter) had a poor ability to accumulate Y^3+^ having a significantly lower adsorption capacity and removal rate of Y^3+^ than the fungal dry powders and the growing process of *Penicillium* sp. ZD28. Moreover, the adsorption quantity by the mycelium pellets was reduced with increasing Y^3+^, showing that high concentrations of yttrium are toxic to cells. At high concentrations of yttrium ions (> 0.6 mM), adsorption during the growth of the fungus was not suitable because the ions can easily precipitate with components of the medium. Therefore, when *Penicillium* sp. ZD28 was used for Y^3+^ adsorption, the high concentration was suitable when using the fungal powder, whilst the low concentration was suitable for adsorption by the growing fungus. In this study, the adsorption of Y^3+^ by the fungal powder was well fitted by the Freundlich, where *R*^*2*^ was equal to 0.9792. And values of n were substantially smaller than 1 suggesting more heterogeneous adsorption sites on the adsorbents (Ahmed et al. 2015). The smaller the *n*, the higher the affinity of the absorbent to the ions (Ji et al. 2010). The *n* in our work was equal to 0.1056, smaller than those of metal ions adsoption by various biological and non-biological materials (Chen et al. 2019; Wei et al. 2019; Mullen et al. 1989). Therefore, *Penicillium* sp. ZD28 has significant potential in the environmental recovery of yttrium ions.

## Data Availability

The data supporting our finding included in the manuscript. Please turn to the corresponding author for all other requests.

## Abbreviations

ICP-MS/MS: inductively coupled plasma tandem mass spectrometry
REEs: rare earth elements
